# The small GTPase Rab5c is a key regulator of trafficking of the CD93/Multimerin-2/β1 integrin complex in endothelial cell adhesion and migration

**DOI:** 10.1186/s12964-019-0375-x

**Published:** 2019-05-28

**Authors:** Stefano Barbera, Federica Nardi, Ines Elia, Giulia Realini, Roberta Lugano, Annalisa Santucci, Gian Marco Tosi, Anna Dimberg, Federico Galvagni, Maurizio Orlandini

**Affiliations:** 1 Department of Biotechnology, Chemistry and Pharmacy, University of Siena, Via A. Moro, 2, 53100 Siena, Italy; 2 Department of Immunology, Genetics and Pathology, Science for Life Laboratory, Rudbeck Laboratory, Uppsala University, SE-751 85 Uppsala, Sweden; 3 Department of Medicine, Surgery and Neuroscience, Ophthalmology Unit, University of Siena, Policlinico “Le Scotte”, Viale Bracci, 53100 Siena, Italy

**Keywords:** Cell polarity, Cell spreading, Moesin, C1qRp

## Abstract

**Background:**

In the endothelium, the single-pass membrane protein CD93, through its interaction with the extracellular matrix protein Multimerin-2, activates signaling pathways that are critical for vascular development and angiogenesis. Trafficking of adhesion molecules through endosomal compartments modulates their signaling output. However, the mechanistic basis coordinating CD93 recycling and its implications for endothelial cell (EC) function remain elusive.

**Methods:**

Human umbilical vein ECs (HUVECs) and human dermal blood ECs (HDBEC) were used in this study. Fluorescence confocal microscopy was employed to follow CD93 retrieval, recycling, and protein colocalization in spreading cells. To better define CD93 trafficking, drug treatments and transfected chimeric wild type and mutant CD93 proteins were used. The scratch assay was used to evaluate cell migration. Gene silencing strategies, flow citometry, and quantification of migratory capability were used to determine the role of Rab5c during CD93 recycling to the cell surface.

**Results:**

Here, we identify the recycling pathway of CD93 following EC adhesion and migration. We show that the cytoplasmic domain of CD93, by its interaction with Moesin and F-actin, is instrumental for CD93 retrieval in adhering and migrating cells and that aberrant endosomal trafficking of CD93 prevents its localization at the leading edge of migration. Moreover, the small GTPase Rab5c turns out to be a key component of the molecular machinery that is able to drive CD93 recycling to the EC surface. Finally, in the Rab5c endosomal compartment CD93 forms a complex with Multimerin-2 and active β1 integrin, which is recycled back to the basolaterally-polarized cell surface by clathrin-independent endocytosis.

**Conclusions:**

Our findings, focusing on the pro-angiogenic receptor CD93, unveil the mechanisms of its polarized trafficking during EC adhesion and migration, opening novel therapeutic opportunities for angiogenic diseases.

**Electronic supplementary material:**

The online version of this article (10.1186/s12964-019-0375-x) contains supplementary material, which is available to authorized users.

## Background

Physiological processes such as development, wound healing, and growth are strictly dependent on vasculature dynamics and require angiogenesis, the branching of new blood vessels from pre-existing ones [1]. As a result, dysregulation of vessel growth has a strong impact on health and contributes to the etiopathogenesis of several disorders, including cancer progression, psoriasis, atherosclerosis, blindness, and infectious disease [2, 3]. Despite several promising drugs have been developed against the main molecules involved in angiogenesis, conventional anti-angiogenic therapies still need to resolve issues such as efficacy, resistance, and toxicity to give long-term meaningful outcomes [4]. Therefore, a better understanding of the mechanisms underlying angiogenesis is mandatory to intervene in pathological processes. Numerous efforts have been made to identify new biomarkers for anti-angiogenic therapy [5]. Recently, molecules belonging to the group XIV family of C-type lectin-like domain (CTLD)-containing proteins have been identified as potential anti-angiogenic targets [6, 7].

CD93 is a single-pass transmembrane protein, which, from the N- to C-terminus, consists of a CTLD domain, five EGF-like repeats, a mucin-like domain, a transmembrane domain, and a cytoplasmic domain. In the juxtamembrane region, CD93 contains a positively charged motif (RKRR), shared with other adhesion molecules, which harbors a binding site for the FERM domain of Moesin, a member of the Ezrin/Radixin/Moesin (ERM) family, involved in the regulation of membrane-cortex interactions and signaling [8, 9]. Since ERM proteins link membrane proteins to actin filaments [10], the interaction between CD93 and Moesin contributes to the reorganization of cytoskeleton, which is essential during cell adhesion and migration [11].

CD93 is predominantly expressed in endothelial cells (ECs) and several data are consistent with a role for CD93 as pro-angiogenic molecule in the vascular endothelium [6, 12–14]. Importantly, CD93 is highly expressed in hyperproliferative ECs of blood vessels within different cancer types and choroidal neovascular membranes of age-related macular degeneration patients [13, 15, 16]. In the activated endothelium, CD93 plays a dual role both operating as adhesion molecule and soluble growth factor [17]. As adhesion molecule, CD93 contributes to EC adhesion and migration through its interaction with Multimerin-2 (MMRN2), an endothelial-specific member of the EDEN family, consistently deposited in the extracellular environment of tumor vasculature [7, 16, 18]. Notably, in tumor angiogenesis the CD93-MMRN2 interaction is required for activation of β1 integrin and formation of a fibrillar fibronectin network [18], which is essential to promote angiogenesis through its continuous turn over and remodeling [19].

Adhesion receptors are endocytosed and recycled through the endosomal pathway and this can fine-tune their spatiotemporal signaling outputs [20]. Moreover, cell adhesion molecule turnover and sorting play a key role during dynamic processes such as cytokinesis, migration, angiogenesis and invasion [21–23] . Within such a framework, Rab GTPases are the major coordinators of endosomal trafficking and regulate vesicle budding, motility, and their tethering to the target compartment, also conferring transport specificity and organelle identity [24, 25]. Here, we investigate the trafficking of CD93 in dynamically active primary ECs to unveil its consequences in angiogenesis regulation. We show the retrieval and recycling of CD93 during EC spreading and migration, identify the cytoplasmic domain of CD93 as a key domain in the endosomal trafficking of CD93 and demonstrate the major role of Rab5c isoform in driving CD93 and β1 integrin delivering to the cell surface to support EC migration.

## Methods

### Cell culture and transfection

Human umbilical vein ECs (HUVECs) and human dermal blood ECs (HDBECs) were purchased from PromoCell (Heidelberg, Germany). Cells were grown on gelatin-coated plates as previously described [18, 26]. For spreading analyses, cells were detached from the culture plate by using enzyme-free cell dissociation buffer (Thermo Fisher Scientific, Waltham, MA, USA), plated on gelatin-coated coverslips, and allowed to spread for different times. Cells at specific phases of spreading were fixed, labeled, and imaged by microscopy. Transient transfection experiments were performed by electroporation as previously described [12].

### DNA constructs

The chimeric construct expressing human CD93 fused to YFP was previously described [12]. The deletion mutant CD93∆C (covering the amino acid residues from 1 to 604 of the human CD93 sequence) fused to YFP was obtained by PCR amplification of a cDNA clone corresponding to the human *CD93* gene, using the following oligonucleotides: 5′-GAGAATTCATGGCCACCTCCATGGG-3′ and 5′-GAGGATCCACCAGTAGCCCCAGAGCC-3′. PCR fragments were cloned into pEYFP-N1 vector (Clontech Labs, Fremont, CA, USA). The construct was confirmed by sequencing.

### Reagents and antibodies

Latrunculin B (Calbiochem-Novabiochem Corp., San Diego, CA, USA) and nocodazole (Sigma-Aldrich, Saint Louis, MO, USA) were used as previously described to disrupt actin and microtubule cytoskeleton integrity, respectively [27]. Cycloheximide (Sigma-Aldrich) was used to inhibit protein synthesis in HUVECs at the concentration of 50 μg/mL.

The following primary antibodies were used: mouse monoclonal anti-CD93 (mAb 4E1) [6], rabbit anti-MMRN2 (generously provided by M. Mongiat), rabbit anti-CD93 (HPA009300, Atlas Antibodies, Bromma, Sweden), mouse anti-CD93 (MBL International Corporation, Woburn, MA, USA), rabbit anti-Giantin, mouse anti-β1 integrin (12G10), and mouse anti-Rab7 (Abcam, Cambridge, UK), rabbit anti-Moesin (Cell Signaling Technology, Danvers, MA, USA), mouse anti-Rab5 (BD Biosciences, Franklin Lakes, NJ, USA), mouse anti-β-actin (Sigma-Aldrich), rabbit anti-CD93 (H-190), mouse anti-COPD (E-12), mouse anti-Sec31A (H-2), mouse anti-β-Adaptin (A-5), mouse anti-Rab5a (E-11), mouse anti-Rab5b (F-9), mouse anti-Rab5c (H-3), mouse anti-β1 integrin (4B7R), mouse anti-Rab11a (D-3), rabbit anti-caveolin-1 (N-20), and mouse anti-MMRN2 (H572) (Santa Cruz Biotechnology, Dallas, TX, USA). Alexa Fluor-488 and -647 phalloidin (Thermo Fisher Scientic) were used for F-actin labeling.

### Immunofluorescence microscopy

Cells were seeded onto gelatin-coated glass coverslips, fixed in 3% paraformaldehyde, and treated as previously described [18, 28]. The secondary antibodies used were conjugated with Alexa Fluor-488 and Alexa Fluor-568 (Thermo Fisher Scientific). Fluorescent images were captured using a Leica TCS SP2 AOBS confocal laser-scanning microscope and overlaid images were produced. A Leica HCX PL APO lbd.BL 63x/1.40 oil objective was used. Fluorochromes and fluorescent proteins were excited at the optimal wave-length ranging from 458 nm to 633 nm and images (512 × 512 resolution) acquired at a scan speed of 400 Hz image lines/sec. Confocal scanner configuration was set as follows: pinhole at 1.0 Airy diameter and line averaging function at 4. To better dissect the labeled cellular structure, some cells were shown as lateral views, corresponding to single *xz* planes. These images were processed as previously described [27]. Cell distribution of exogenous CD93 proteins and protein localization at the migrating front were measured using NIS-Elements image analysis software (Nikon Instruments, Melville, NY, USA). For protein quantification at the migrating front, an area of 20 μm distance from the leading edge was chosen, and a minimum of 5 images of the migrating front area per condition were used. Similarly, exogenous CD93 was quantified in the migrating front area of single transfected cells. The quantitative colocalization analyses were performed pre-processing the images and using ImageJ 2.0 and the JACoP plug-in to determine Manders coefficients and Costes randomization values as previously described [29]. Costes *P* values ≥95% were considered as a colocalization signal. To show colocalization events by white dots, images were generated using ImageJ and the Colocalization plug-in.

### RNA interference, immunoblotting analysis, and flow cytometry

Silencing experiments were performed using pLKO.1 retroviral vectors from the MISSION shRNA Library (Sigma-Aldrich) expressing specific shRNAs for human Rab5c (SHCLNG-NM_004583, clone TCRN0000380031, referred to as 31; clone TCRN0000072933, referred to as 33) and human CD93 (clone TRCN0000029085) [6]. Recombinant lentiviruses were produced and used for infection experiments as previously described [28]. Immunoblotting experiments were performed as previously described [30]. Flow cytometry analysis was performed as previously described with slight modifications [31]. Briefly, living cells were detached from the culture plate using enzyme-free cell dissociation buffer, incubated with an anti-CD93 (mAb 4E1) antibody followed by an anti-mouse Alexa Fluor 488-conjugated secondary antibody, and analyzed on GUAVA flow cytometer (Merck Millipore, Burlington, MA, USA).

### Wound healing assay

The scratch test was performed as previously described with slight modifications [16]. Briefly, transfected or lentivirus transduced HUVECs were seeded on glass coverslips in a 24-well plate and cultured until they reached confluency. A straight scratch was created in the monolayer using a sterile pipette tip. The cultures were washed with PBS and grown in complete medium. Bright-field images were captured using an inverted microscope equipped with a digital camera. For each condition, images were acquired at three different positions along the scratch and a representative field was shown. For immunofluorescence analysis, after the scratch cells were washed with PBS, grown in complete medium for 5 h, fixed in 3% paraformaldehyde, and subjected to immunofluorescence analysis.

### Statistical analysis

In this study, the data analysis was performed using Prism 6 software (Graphpad Software, La Jolla, CA, USA). Differences between samples were estimated using the two-tailed student’s *t*-test or 2-way ANOVA followed by Dunnett’s test. Values were expressed as the mean ± SD and *P* < 0.05 was considered statistically significant.

## Results

### In early spreading ECs, CD93 is channeled to the actin-rich apical bud

**Fig. 1:**
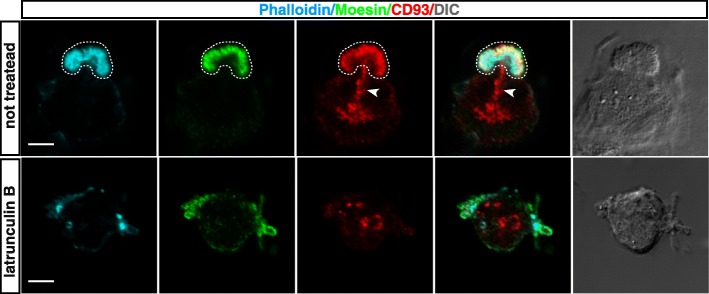
During the initial phases of spreading, CD93 is sorted towards the actin-rich apical bud. HUVECs were pretreated or not for 30 min with latrunculin B, then they were detached from the plate, resuspended in complete growth medium, and plated on the substrate in the presence or not of latrunculin B. 5 min after plating, cells were fixed and analyzed by immunofluorescence using phalloidin, anti-Moesin, and anti-CD93 antibodies. The apical bud is delimited by a dotted line. Arrowheads indicate small vesicles beneath the apical bud. Differential interference contrast (DIC) images of stained cells are shown. Scale bars represent 5 μm

During the early phases of EC adhesion, the cooperation between F-actin and Moesin leads to the assembly of an actin-rich apical domain (also known as apical bud or apical membrane insertion site) that drives apical-basolateral polarization [27, 32]. Since Moesin orchestrates cytoskeletal remodeling and links transmembrane CD93 to the actin cytoskeleton [11], we questioned whether the localization of CD93 was polarized during spreading of human primary ECs. To address this issue, exponentially growing HUVECs were detached from the plate, allowed to adhere on the substrate, and analyzed by immunofluorescence. Interestingly, at early degrees of cell spreading CD93 showed a pattern of localization similar to Moesin, being mainly confined into the apical bud and colocalizing with F-actin (Fig. 1, upper panels). Moreover, CD93 was also localized in small vesicles clustered directly beneath the apical bud (Fig. 1, upper panels), which in turn colocalized with caveolin-1 (Additional file 1: Figure S1), a key player in promoting EC polarization [33]. To assess whether such distribution depended on F-actin, ECs were let to settle on the substrate in the presence of latrunculin B, which disrupts microfilament organization. Consistent with our previous findings [27], disruption of the F-actin cytoskeleton resulted in the impairment of apical bud structure, thus hampering cell polarity and eliciting a random distribution of CD93 throughout the adhering cell (Fig. 1, lower panels).

**Fig. 2:**
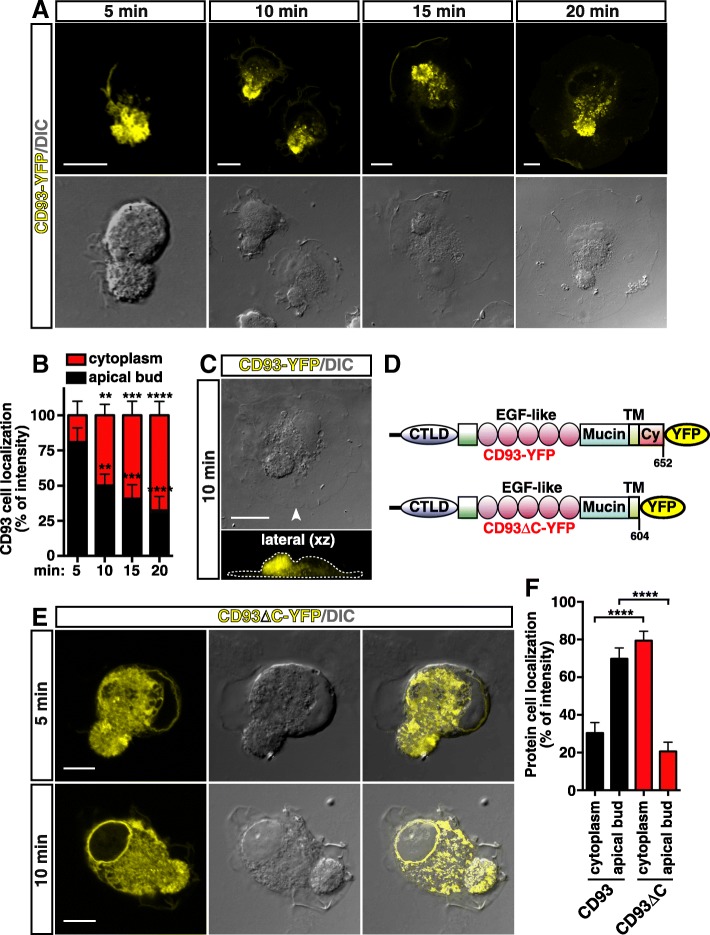
Retrieval of CD93 is regulated through its cytoplasmic domain. **a**: Time course image analysis of CD93 localization during cell spreading. CD93-YFP was transiently transfected into HUVECs. Cells were detached from the plate, resuspended in complete growth medium, plated on the substrate, and fixed at different spreading times as indicated. Exogenous CD93 was imaged as yellow. Scale bars, 10 μm. **b**: Quantification of exogenous CD93 levels in the apical bud and cytoplasm of ECs treated as in **a**. Bars represent the percentage of total fluorescence intensity/area. Data are presented as the mean/cell ± SD of three independent experiments (*n* = 3 cells per group). **P < 0.01, ***P < 0.001, ****P < 0.0001 vs. 5 min; 2-way ANOVA with Dunnett’s post-test. **c**: Periodic *z*-series of cells treated as in **a** were acquired at 10 min after plating to generate a vertical section (lateral *xz*, 90 *z*-sections of 115 nm each). The corresponding cell boundary is indicated by a dotted line. An arrowhead indicates the point of view of the lateral *xz* image. Scale bar, 15 μm. **d**: The schematic diagram illustrates CD93 wild type (CD93-YFP) and the deletion mutant (CD93∆C-YFP) lacking the cytoplasmic domain fused with YFP. The deleted amino acid residues are indicated. CTLD, C-type lectin-like domain; EGF-like, Epidermal Growth Factor repeats; Mucin, Mucin-like domain; TM, transmembrane domain; Cy, cytoplasmic domain. **e**: CD93∆C-YFP deletion mutant was transiently transfected into HUVECs. Cells were detached from the plate, resuspended in complete growth medium, plated on the substrate, and fixed at early phases of spreading (from 5 to 10 min). Exogenous CD93∆C-YFP was imaged as yellow. Scale bars, 8 μm. Merged and DIC images are shown. **f**: Quantification of exogenous wild type and mutant CD93 levels in the apical bud and cytoplasm of transfected cells as in **a** and **e**. Bars represent the percentage of total fluorescence intensity/area. Data are presented as the mean/cell ± SD of three independent experiments (*n* = 15 cells per group). ****P < 0.0001; unpaired *t*-test

Next, to better explore the trafficking of CD93 in spreading cells, HUVECs were transfected with an expression vector encoding wild type CD93 fused with YFP at its C-terminus [12]. Fluorescent confocal microscopy was employed to analyze cells fixed at different stages of cell flattening against the substrate. The tagged wild type protein behaved as the endogenous CD93 and it resulted localized to the apical bud (Fig. 2a, 5 min). Importantly, confocal microscopy analysis at increasing times of cell spreading showed that small vesicles containing CD93-YFP moved from the apical bud towards the middle of the cell (Fig. 2a, 10 min, 15 min, and 20 min, quantified in Fig. 2b). This localization was further corroborated by confocal *xz* scans that created a cross-sectional view through a spreading cell (Fig. 2c). Since the cytoplasmic domain of CD93 interacts with the FERM domain of Moesin [11], we questioned whether the cytoplasmic domain was involved in the apical bud localization of CD93. To address this possibility, we generated a YFP-tagged CD93 deletion mutant (CD93∆C-YFP) lacking the cytoplasmic tail and transfected it into HUVECs (Fig. 2d). Imaging of cells fixed at early attachment stages showed that CD93∆C-YFP was diffusely distributed throughout the cytoplasm and the small vesicles containing the deletion mutant were not polarized (Fig. 2e). These observations were further substantiated by quantitative analysis of exogenous CD93 and CD93∆C levels in the apical bud and cytoplasm of early spreading cells (Fig. 2f). Taken together, these findings indicate that in ECs undergoing early spreading, the cytoplasmic domain of CD93 through interaction with Moesin and F-actin drives the localization of CD93 to the polarizing apical bud and its endocytic movements towards the middle of the cell.

### F-actin is involved in the endocytic trafficking of CD93

**Fig. 3:**
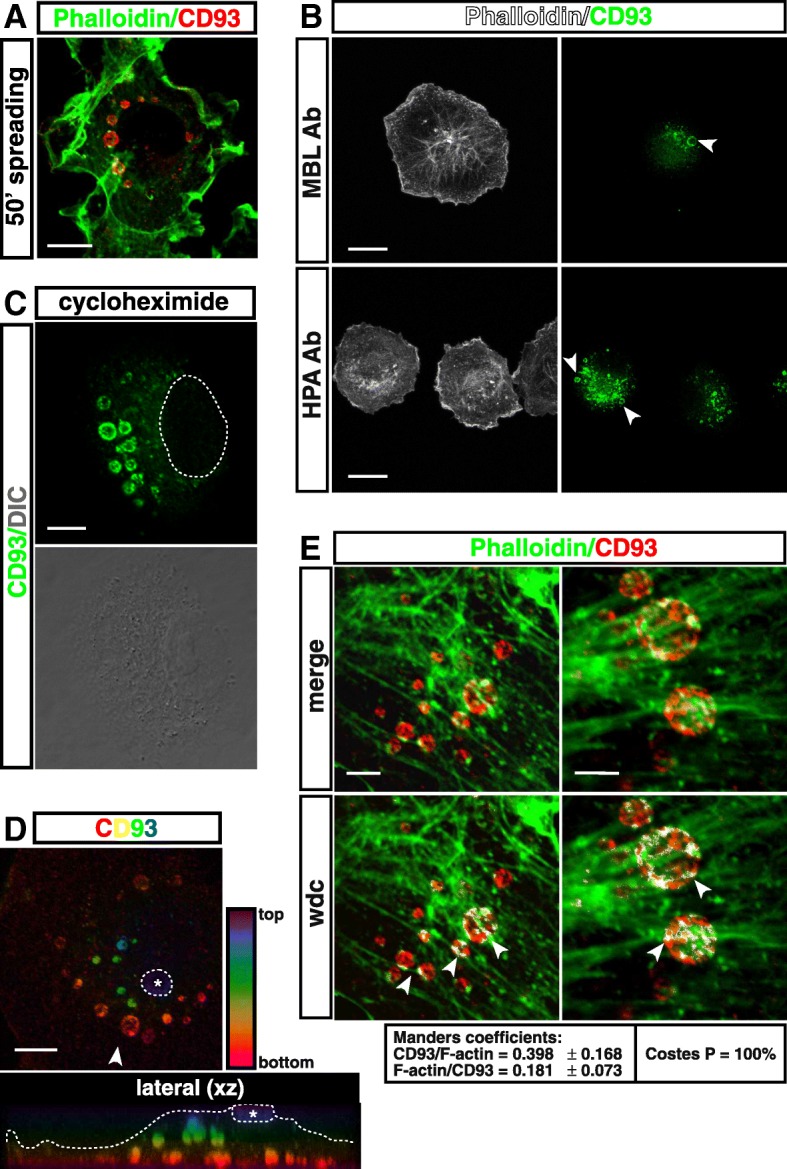
CD93 is recycled via large vesicles moving along F-actin cables. **a**: Exponentially growing HUVECs were detached from the plate, resuspended in complete growth medium, plated on the substrate, and fixed at late phases of spreading (from 30 to 50 min). Cells were analyzed by immunofluorescence using phalloidin and an anti-CD93 (4E1) antibody. Scale bar, 11 μm. **b**: Exponentially growing HDBECs were treated as in **a**. Cells were analyzed by immunofluorescence using phalloidin and two different anti-CD93 antibodies as indicated. Arrowheads indicate CD93^+ve^ vesicles. Images were acquired using a Leica SP8 confocal microscope equipped with Leica White Light Laser, notch filters (488 nm, 568 nm, and 647 nm) and a HC PL APO CS2 63x/1.40 oil objective. Images were taken in 1024 × 1024 format, speed 400 Hz, and pinhole set at 1.0 Airy diameter. Scale bars, 20 μm. **c**: HUVECs were pretreated for 4 h with 50 μg/mL cycloheximide, then they were detached from the plate, resuspended in complete growth medium, and plated on the substrate in the presence of cycloheximide. Cells were analyzed by immunofluorescence at late phases of spreading using anti-CD93 antibodies. DIC image of stained cells is shown. The dotted line indicates nucleus boundary. Scale bar, 8 μm. **d**: Periodic *z*-series of ECs treated as in **a** were acquired at 50 min after plating to generate a color-coded projection such that the basal cell surface, which adheres to the substrate is red and the apical cell surface is violet. A vertical section (lateral *xz*, 47 *z*-sections of 115 nm each) obtained from the same images is shown. A dotted ring containing an asterisk indicates apical bud boundary. In the *xz* view, a dotted line indicates the cell boundary and an arrowhead the point of view of the image. Scale bar, 8 μm. **e**: Details of late spreading HUVECs. Cells treated as in **a** were stained for F-actin and CD93. Overlay of stained cells and white dot colocalization (wdc) images are shown. Arrowheads indicate colocalization between CD93^+ve^ vesicles and actin cables. Manders and Costes quantitative analyses of CD93 colocalization with F-actin and vice versa are shown (*n* = 8 cells). Scale bars are 4 μm

As EC flattening against the substrate increased, we observed the appearance of numerous, large and round-shape CD93-positive (CD93^+ve^) vesicles arranged in the perinuclear region (Fig. 3a). Of note, also in spreading HDBECs and with the use of different antibodies against CD93, confocal microscopy analysis revealed the appearance of large round-shape CD93^+ve^ vesicles (Fig. 3b), suggesting that the formation of large CD93^+ve^ recycling vesicles is a typical trait in spreading primary ECs. HUVECs treated with cycloheximide showed regular formation of CD93^+ve^ vesicles during the late spreading phases (Fig. 3c), suggesting that these vesicles contain a recycled pool of pre-existing CD93 and their origin is not due to new protein synthesis. Furthermore, confocal color-coded *xz* sectioning localized the smaller CD93^+ve^ vesicles near and around the apical bud and the largest ones close to the basolateral membrane (Fig. 3d), suggesting that the CD93^+ve^ vesicles move from the apical bud towards the cell-substrate interface. Importantly, high-resolution fluorescence confocal microscopy analysis of CD93^+ve^ vesicles showed their alignment with the actin cables (Fig. 3e). Since actin dynamics ensure efficient endocytosis and direct the trafficking of vesicles to the cell surface [34], we hypothesized a role for F-actin in the transport of CD93^+ve^ vesicles. To explore this hypothesis, ECs were let to spread in the presence of latrunculin B. Accordingly, addition of this drug to the culture medium impaired the formation of CD93^+ve^ vesicles and CD93 resulted mislocalized in several cytosolic regions (Additional file 1: Figure S2). By contrast, when the microtubule cytoskeleton was disrupted by nocodazole, no effects were observed on the formation and localization of CD93^+ve^ vesicles (Additional file 1: Figure S3). Since recent studies have demonstrated that internalized adhesion molecules may transit through the *trans*-Golgi network (TGN) before being delivered to the cell surface [19], we asked whether also CD93 could be involved in the route from or to the Golgi apparatus. To address this possibility, we performed immunofluorescence analyses on early and late spreading HUVECs by staining coatomers involved in the secretory pathway, such as COPI, COPII, and clathrin. However, protein involved in the secretory pathway did not show significant colocalization with CD93 (Additional file 1: Figure S4). Altogether, these data indicate that in spreading ECs, endocytosed CD93 is directly recycled back to the cell-substrate interface via vesicles moving along F-actin cables.

### MMRN2 and β1 integrin are recycled together with CD93

**Fig. 4:**
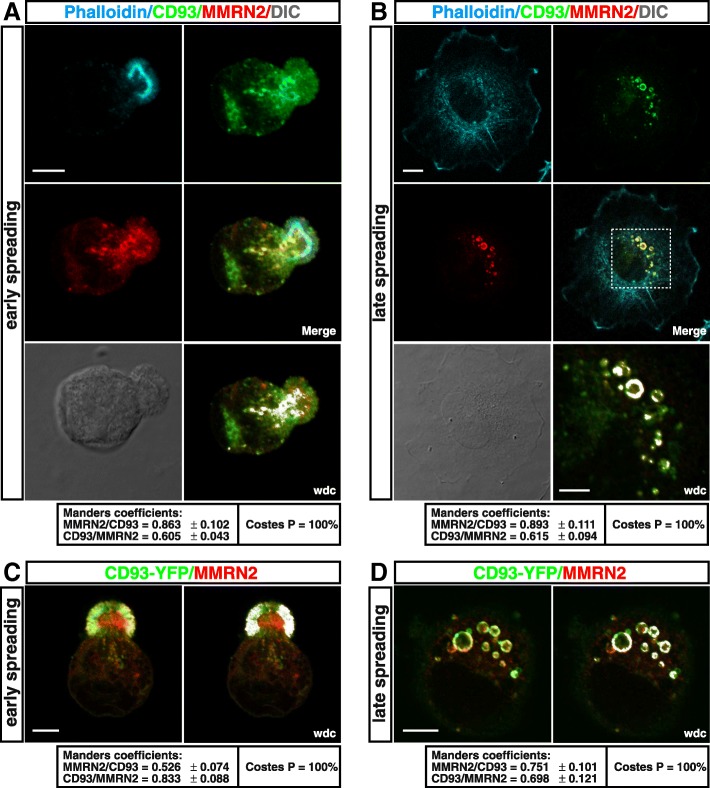
CD93 colocalizes with MMRN2 in endocytic vesicles. The CD93-YFP construct was transfected or not into HUVECs. Cells were detached from the plate, resuspended in complete growth medium, plated, fixed at different times after plating, and analyzed by confocal microscopy. **a**, **b**: Untransfected ECs at early and late phases of spreading were stained using phalloidin, anti-CD93, and anti-MMRN2 antibodies. Merged, DIC, and wdc images between CD93 and MMRN2 are shown. Scale bars, 7 μm. In **b**, the wdc picture shows a high magnification of the CD93^+ve^ vesicles displayed into the dashed square. Scale bar, 4 μm. **c**, **d**: Transfected early and late spreading cells were stained for MMRN2. Exogenous CD93 was imaged as green. Overlay of stained cells and wdc images are shown. Scale bars, 7 μm. For each condition, Manders and Costes quantitative analyses of MMRN2 colocalization with CD93 and vice versa are reported (*n* = 5–7 cells)

**Fig. 5:**
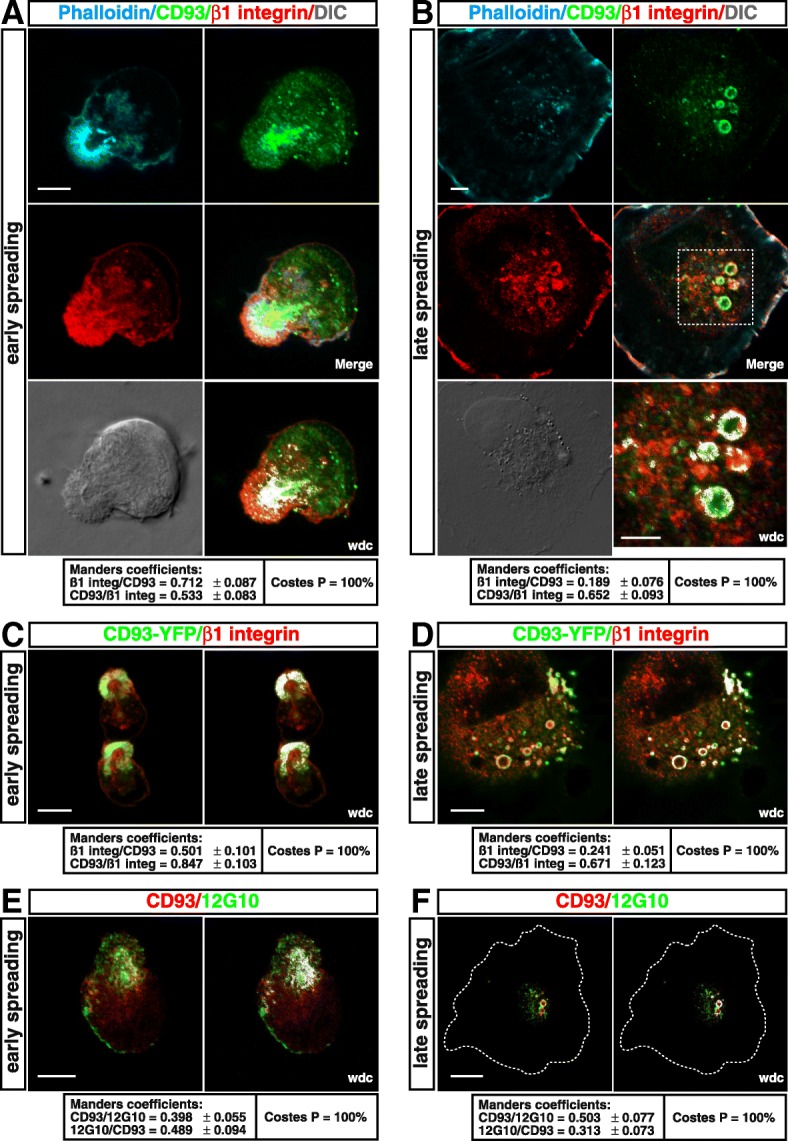
The CD93-MMRN2 complex is recycled in association with β1 integrin. The CD93-YFP construct was transfected or not into HUVECs. Cells were detached from the plate, resuspended in complete growth medium, plated, fixed at different times after plating, and imaged by immunofluorescence. **a**, **b**: Untransfected cells at early and late phases of spreading were stained using phalloidin, anti-CD93 and anti-β1 integrin antibodies. Merged, DIC, and wdc images between CD93 and β1 integrin are shown. In **b**, the wdc picture shows a high magnification of the CD93^+ve^ vesicles visible into the dotted square. Scale bars, 6 μm (**a**), 4 μm (**b**). **c**, **d**: Transfected early and late spreading ECs were stained for β1 integrin. Exogenous CD93 was imaged as green. Overlay of stained cells and wdc images are shown. Scale bars, 8 μm (**c**) and 7 μm (**d**). **e**, **f**: Immunofluorescent staining of CD93 and active β1 integrin (12G10) in untransfected cells at early and late phases of attachment to the substrate. Merged and wdc images are shown. In **f**, dotted lines indicate cell boundary. Scale bars, 8 μm (**e**) and 13 μm (**f**). For each condition, Manders and Costes quantitative analyses of β1 integrin colocalization with CD93 and vice versa are reported (*n* = 4–8 cells)

Several adhesion molecules are often internalized together with their ligands and other associated proteins in functional domains on the membrane surface. Such associations allow the coordination of ligand-receptor recycling and degradation thus controlling their bioavailability and function [35]. Hence, we sought to investigate whether endocytosed CD93 was associated with its ligand MMRN2 in internalized vesicles. To this end, endogenous CD93 and transfected CD93-YFP were analyzed by fluorescence confocal microscopy at different degrees of cell adhesion. Interestingly, both endogenous and exogenous CD93 were colocalized with MMRN2 at early and late spreading phases (Fig. 4). Such colocalization was also observed in early and late spreading HDBECs (Additional file 1: Figure S5). Since CD93 and MMRN2 cooperate to stabilize α5β1 integrin and β integrin subunits are mainly associated with regulatory and signal transducing proteins [18, 36], we asked whether β1 integrin was also internalized in association with the CD93-MMRN2 complex during cell spreading. Notably, both endogenous and exogenous CD93 were colocalized with β1 integrin, which followed the same endocytic route of CD93 during early and late cell spreading (Fig. 5a-d). Moreover, internalized β1 integrin resulted partly bound to ECM, as assessed by immunofluorescent staining using an antibody that specifically recognizes the active conformation of β1 integrin (Fig. 5e and f). Taken together, these findings indicate that during EC adhesion, endocytosed CD93 forms an active complex with MMRN2 and β1 integrin and the whole complex is retrieved and recycled directly back to the plasma membrane.

### Rab5c promotes CD93 recycling

**Fig. 6:**
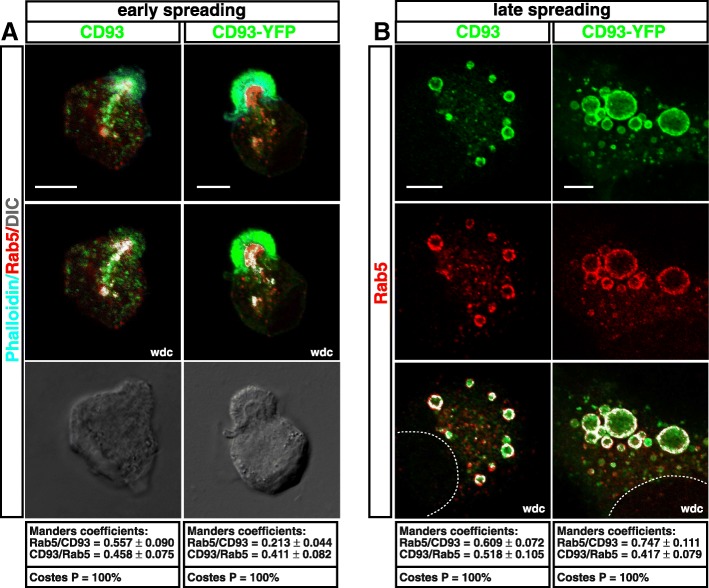
CD93 colocalizes with the small GTPase Rab5. HUVECs were transfected or not with CD93-YFP, then they were detached from the plate, resuspended in complete growth medium, plated on the substrate, fixed at early (**a**) and late (**b**) degrees of cell spreading, and subjected to immunofluorescence analysis. **a**: Untransfected cells (CD93) were stained with phalloidin, anti-Rab5, and anti-CD93 antibodies. Transfected cells (CD93-YFP) were stained with phalloidin and anti-Rab5 antibodies. Scale bars, 7 μm. **b**: Untransfected cells (CD93) were stained with anti-CD93 and anti-Rab5 antibodies. Transfected cells (CD93-YFP) were stained for Rab5. A dotted line indicates nucleus boundary. Scale bars, 7 μm. In transfected cells, exogenous CD93 was imaged as green. Merged, DIC, and wdc images between CD93 and Rab5 are shown. For each condition, Manders and Costes quantitative analyses of Rab5 colocalization with CD93 and vice versa are reported (*n* = 5–8 cells)

**Fig. 7:**
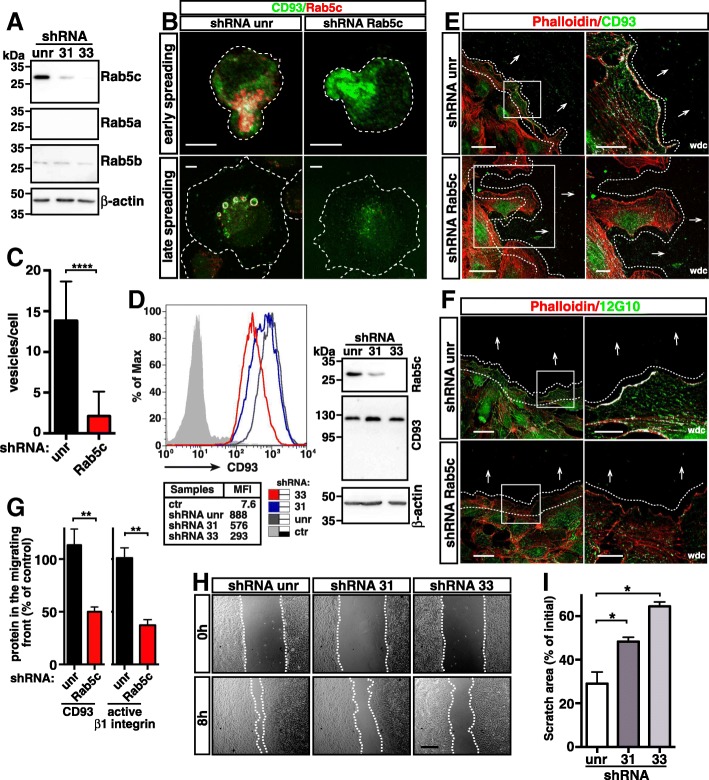
Rab5c modulates CD93-mediated EC motility. HUVECs were transduced with lentiviral particles expressing unrelated (unr) or Rab5c shRNAs (clone 31 and 33). **a**: Cell lysates from shRNA expressing ECs were analyzed by Western blotting using antibodies against Rab5a, Rab5b, or Rab5c. Anti-β-actin antibodies were used to confirm equal loading. **b**: Representative images of control (shRNA unr) and Rab5c-silenced (shRNA Rab5c, clone 33) HUVECs at early and late phases of spreading. Cells were analyzed by confocal microscopy using anti-CD93 and anti-Rab5c antibodies. Overlay of stained cells is shown. Dashed lines indicate cell boundaries. Scale bars, 6 μm. **c**: Quantitative analysis of CD93^+ve^ vesicles per cell from late spreading ECs treated as in **b**. The vesicle numbers represent the mean ± SD of three independent experiments (*n* = 20 cells). ****P < 0.0001; unpaired *t*-test. **d**: Flow cytometry analysis of CD93 plasma membrane levels in control (unr) and Rab5c-silenced (clones 31 and 33) HUVECs. Cells stained only with the secondary antibody are shown (ctr). The mean fluorescence intensity (MFI) is reported. Total cell extracts from the same shRNA expressing cells were analyzed by Western blotting using anti-CD93 and anti-Rab5c antibodies. Anti-β-actin antibodies were used to confirm equal loading. **e**, **f**: Representative images of the confocal microscopy analyses of control (shRNA unr) and Rab5c-silenced (shRNA Rab5c, clone 33) HUVECs at 5 h after production of a double-sided scratch in the cell monolayer. Phalloidin and anti-CD93 (**e**) or anti-β1 integrin (12G10) (**f**) antibodies were used to image cells. Arrows indicate direction of migration and dotted lines indicate the migrating front. Overlay of stained cells is shown. Scale bars, 40 μm. Magnifications of the squared areas are shown as wdc images. In wdc images, scale bars are 15 μm. In **b**, **e**, and **f**, same results were obtained when using the clone 31 for Rab5c knockdown. **g**: Quantification of CD93 and active β1 integrin along the migrating front areas indicated by dotted lines in **e** and **f**. Bars represent the percentage of fluorescence intensity of the control. Data are presented as the mean ± SD of three independent experiments (*n* = 5 different areas along the migrating edge). **P < 0.01; unpaired *t*-test. **h**: Representative images of wound closure in HUVECs transduced with lentiviral particles expressing control (unr) or Rab5c shRNAs. Cells were photographed at 0 and 8 h. Scale bar, 100 μm. **i**: The percentage of scratch area was calculated from images acquired at time 0 and 8 h following the wound. Analyses were performed using ImageJ. The graph represents the means ± SD, *n* = 6 images per condition pooled from two independent experiments. *P < 0.05; unpaired *t*-test

To investigate the nature of CD93 recycling in spreading ECs, we focused on Rab GTPases, which have been demonstrated to regulate endocytic trafficking and contribute to the structural and functional identity of intracellular organelles [25]. To this end, HUVECs were transfected or not with CD93-YFP, fixed, and stained for Rab5, Rab7, and Rab11 at different degrees of attachment to the substrate. CD93^+ve^ vesicles formed during early and late spreading showed a strong colocalization of endogenous and exogenous CD93 with the early endosomal marker Rab5 (Fig. 6), but not with Rab7 and only slightly with Rab11 in the large vesicles (Additional file 1: Figure S6), suggesting that Rab11 contributes to the transition of CD93^+ve^ vesicles from early to recycling endosomes [37]. We hence infer that Rab5 is involved in the regulation of CD93 recycling. Since Rab5 has three isoforms (Rab5a, b, c) that cooperate in the regulation of endocytosis in eukaryotic cells [38], we first investigated whether all isoforms were expressed in HUVECs. Interestingly, only a strong Rab5c signal was detected by both immunofluorescence and immunoblotting analyses (Additional file 1: Figure S7). Next, to directly explore the role of Rab5c in CD93 recycling, we sought to impair Rab5c expression in HUVECs by lentiviral constructs expressing two independent shRNAs (clones 31 and 33). ECs transduced with lentiviruses expressing both Rab5c shRNAs showed a strong decrease in Rab5c protein levels as compared to ECs transduced with a lentivirus expressing an unrelated shRNA (Fig. 7a). Importantly, the silencing of Rab5c did not increase the expression pattern of the other Rab5 isoforms (Fig. 7a), suggesting that in HUVECs Rab5 isoforms do not undergo evident genetic compensation in response to gene knockdown [39]. Moreover, confocal microscopy analysis on Rab5c-silenced HUVECs showed that in early spreading cells, CD93 was regularly distributed in small vesicles beneath the apical bud (Fig. 7b, upper panels). By contrast, in late spreading ECs, confocal microscopy and quantitative analyses showed that Rab5c downregulation resulted in a remarkable disappearance of large CD93^+ve^ vesicles (Fig. 7b lower panels, and 7c), suggesting that Rab5c is involved in the intracellular trafficking and size increase of CD93^+ve^ vesicles but not in the internalization of CD93 from the apical bud. To evaluate further the involvement of Rab5c in the regulation of CD93 recycling, we analyzed plasma membrane and total cellular levels of CD93 in control and Rab5c-silenced ECs by flow cytometry and Western blotting. Notably, we observed that, although the total CD93 cellular levels remained unchanged, less CD93 protein was exposed on the plasma surface of Rab5c-silenced cells in comparison to control (Fig. 7d).

Next, to assess the role of Rab5c in the trafficking of CD93 during EC migration, we employed an in vitro wound healing assay and used confocal microscopy to detect the subcellular localization of CD93 in the leading edge of migrating cells. Similarly to what has been previously reported for HDBECs [18], CD93 was strongly localized in the migrating edge of HUVECs transduced with a lentivirus expressing an unrelated shRNA (Fig. 7e, upper panels). On the contrary, Rab5c silencing dramatically impaired the localization of CD93 in the extending lamellipodia of the migrating front (Fig. 7e lower panels, quantified in Fig. 7g). These findings were further substantiated by internalization assays on migrating control and Rab5c-silenced ECs (Additional file 1: Figure S8). Importantly, in line with the role of CD93 in modulating β1 integrin activation during cell migration [18], Rab5c silencing strongly reduced the amount of β1 integrin as well as its active form at the leading edge of migration (Additional file 1: Figure S9 and Fig. 7f, quantified in Fig. 7g). In keeping with the above results, knocking down Rab5c reduced the ability of HUVECs to migrate in wound closure assays (Fig. 7h and i), indicating that in ECs the Rab5c isoform orchestrates the recycling of endocytosed CD93 involved in the regulation of β1 integrin activation during cell migration.

### In adhering and migrating ECs, the cytoplasmic domain of CD93 is critical for CD93 recycling

**Fig. 8:**
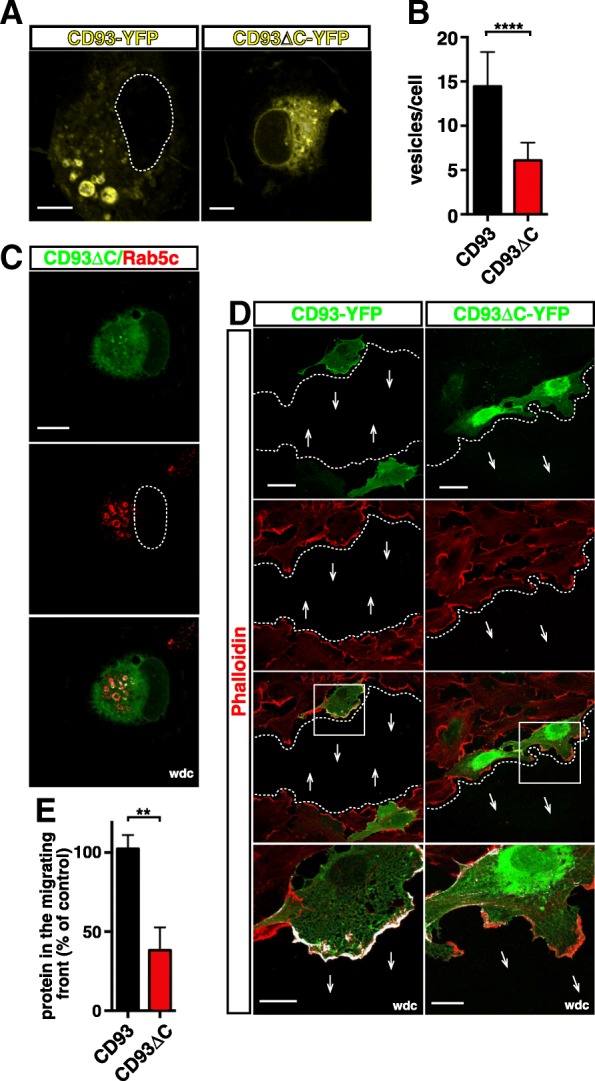
During EC adhesion and migration, CD93 is recycled through its intracellular domain. YFP-tagged CD93 or CD93∆C deletion mutant were transfected into HUVECs. **a**: Cells were detached from the plate, resuspended in complete growth medium, plated on the substrate, fixed at late phases of spreading, and analyzed by confocal microscopy. Exogenous proteins were imaged as yellow. Dotted lines indicate nucleus boundary. Scale bars are 7 μm. **b**: Quantitative analysis of CD93^+ve^ vesicles per cell from ECs treated as in **a**. The vesicle numbers represent the mean ± SD of three independent experiments (*n* = 20 cells). ****P < 0.0001; unpaired *t*-test. **c**: Representative images of transfected HUVECs fixed at late phases of spreading. Cells were analyzed by confocal microscopy using anti-Rab5c antibodies. Exogenous CD93∆C was imaged as green. A dashed line indicates nucleus boundary. A wdc image between CD93∆C and Rab5c is shown. Scale bar, 15 μm. **d**: F-actin and wild type or mutant CD93 were imaged at 5 h after production of a double-sided scratch in the cell monolayer. Arrows indicate direction of migration and dotted lines indicate the migrating front. Overlay of stained cells is shown. Scale bar, 40 μm. High-magnification pictures of ECs in the migrating front within the squared area are shown as wdc images. In wdc images, scale bars are 15 μm. **e**: Quantification of CD93 and CD93∆C along the migrating front of transfected cells. Bars represent the percentage of fluorescence intensity of the control. Data are presented as the mean ± SD of three independent experiments (*n* = 7 transfected cells along the migrating edge). **P < 0.01; unpaired *t*-test

Since the cytoplasmic tail of CD93 drives the movements of CD93 towards the polarizing apical bud (Fig. 2d), we hypothesized its involvement also in the transport of CD93 to the cell-substrate interface. To explore this hypothesis, HUVECs were transfected with CD93-YFP or CD93∆C-YFP constructs and the distribution of the chimeric proteins was compared by immunofluorescence analyses at late degrees of cell adhesion. As shown in Fig. 8a (left panel), exogenous CD93 was specifically localized in large vesicles such as the endogenous protein. On the other hand, the CD93∆C tagged protein showed a diffuse and punctate pattern throughout the cytoplasm and the CD93^+ve^ vesicles showed a strong reduction in size (Fig. 8a, right panel). These observations were sustained by quantitative analysis of CD93^+ve^ vesicle number, highlighting its statistically significant decrease in cells transfected with the deletion mutant in comparison to cells transfected with wild type CD93 (Fig. 8b). Of note, neither the presence of CD93∆C nor the downregulation of CD93 impaired the formation of large Rab5c^+ve^ vesicles (Fig. 8c and Additional file 1: Figure S10), suggesting that the cytoplasmic random distribution of CD93∆C is not due to destruction of subcellular structures.

To investigate the role of the cytoplasmic tail in the recycling of CD93 during EC migration, we transfected HUVECs with CD93-YFP or CD93∆C-YFP constructs, used an in vitro wound healing assay and assessed the subcellular localization of exogenous proteins by confocal microscopy and quantitative analysis of protein localization at the migrating front. While exogenous wild type CD93 was localized in the leading edge of migration, where ECs extended lamellipodia, only low levels of CD93∆C were observed in the extending lamellipodia of the migrating front, being mainly accumulated in the perinuclear region (Fig. 8d, quantified in Fig. 8e). Altogether, these results suggest that the C-terminus of CD93 controls its retrieval and recycling to the cell surface, and as a result, it regulates cell adhesion and movements of migrating ECs.

## Discussion

In developing blood vessels, ECs exhibit polarity in several axes and polarization of endothelia is essential for morphogenesis of the vascular tree [40]. The establishment of polarity is regulated by the spatiotemporal coordination of different signaling pathways that modulates cytoskeletal remodeling and vesicle trafficking to specify membrane domains [1]. Understanding the underlying mechanisms of EC polarity may help to elucidate the pathophysiology of angiogenic diseases and to improve their treatment [20]. Hence, in this study we investigated the polarized trafficking of CD93, an EC adhesion molecule recently identified as a key regulator of neo-vascularization processes [6, 13, 15].

During cell spreading, a process strictly connected to migration and mitosis, ECs undergo apical-basolateral polarization [41]. We found that at onset of EC spreading CD93 is completely localized to the apical bud, a membrane Moesin-rich domain branching out linear actin cables and able to mediate polarized trafficking [27, 32]. Accordingly, both drug disruption of the actin cytoskeleton and deletion of the CD93 cytoplasmic domain containing the binding site to Moesin severely impair CD93 localization to the apical bud, indicating that hampering the interaction with F-actin prevents a proper CD93 redistribution in the early phases of cell attachment to the substrate. Indeed, it has been shown that Moesin, acting as a bridge between actin microfilaments and plasma membrane, alters cell morphology, motility, and cell polarity [9, 42]. Moesin is maintained in a quiescent state by a masked closed conformation and it is activated following threonine phosphorylation in its C-terminal domain. This phosphorylation is closely associated with cytoskeletal rearrangement and there is now evidence that Moesin phosphorylation can also contribute to enhanced angiogenesis [42, 43]. Of note, in the apical bud, most of Moesin is phosphorylated [27], suggesting that, in early spreading ECs, activated Moesin and F-actin act synergistically to localize CD93 to the apical bud.

Polarized membrane transport assists continuous reorganization of the cell membrane and it is involved in maintaining polarity and recycling of essential components in spreading and motile cells [20, 44]. Consistent with this, as soon as EC spreading proceeds, we first observed CD93 in cytoplasmic vesicles clustered directly beneath the apical bud and colocalizing with caveolin-1 but not with β-Adaptin, a component of the AP1 and AP2 adapter complexes, indicating that clathrin-independent endocytosis generates CD93^+ve^ vesicles. Of note, clathrin-independent endocytosis, mediated by caveolae, has been linked to cellular spreading, cell polarization, and modulation of intercellular signaling [45]. Next, departing from the apical bud, these CD93^+ve^ vesicles grow in size and, in tight association with F-actin, move towards the cell-substrate interface where they release their cargo. Upon endocytosis, apical and basolateral cargoes enter spatially distinct early or basolateral endosomes, which typically carry Rab5, a small GTPase also involved in the process of homotypic fusion between early endosomes [46, 47]. In line with these findings, we showed that endocytic CD93^+ve^ vesicles strongly colocalize with Rab5c and depletion of Rab5c causes their decrease in size. Importantly, despite bidirectional ER-Golgi transport has emerged as a key regulator of apical transport and lumen morphogenesis [48], CD93 recycling does not transit through the TGN and thus is not tethered to the secretory pathway as previously shown for other adhesion molecules [19]. Furthermore, we found that endocytic CD93^+ve^ vesicles carry MMRN2 and active β1 integrin, suggesting that following cell attachment to the substrate, the internalized CD93/MMRN2/β1 integrin active complex is directly recycled back to the polarized cell surface. Consistently with this assumption, in ECs the reciprocal interaction between CD93, MMRN2 and β1 integrin is essential for activation of β1 integrin [18], an adhesion molecule emerged also as a regulator of EC polarity and lumen formation in the developing vasculature [49]. In this respect, for some cargo the choice of a direct or indirect sorting pathway to the cell surface depends on the degree to which polarity has been established [47]. Here, we showed how in spreading ECs CD93 is retrieved and recycled directly back to the plasma membrane together with MMRN2 and consequently active β1 integrin. This endocytic pathway of CD93, also sustained by previous studies showing that integrins and their ligands exhibit a high traffic-dependent turnover within adhesion sites and that Rab5 controls β1 integrin internalization [50] [51], represents an ideal strategy for enabling cells to sense and rapidly respond to chemical cues from the surrounding extracellular environment.

Since Rab5 is a signaling protein involved in actin remodeling triggered by receptor tyrosine kinases [52], identification of Rab-mediated steps in the CD93 endocytic route argues for a network of interactions that impact on vascular features such as migration. Rab5 is a master regulator of early endosome biogenesis [53]. However, Rab5 has three isoforms, Rab5a, b, c, that have overlapping yet distinct functions [25]. Interestingly, we found that Rab5c is the preferentially expressed isoform in HUVECs and that its depletion causes EC defective migration towards open wound space in a scratch assay by preventing the delivering of CD93 and active β1 integrin to the extending lamellipodia of the migrating front. Intriguingly, recent reports indicate that in HeLa cells Rab5c selectively regulates cell motility and cytoskeletal dynamics and that Rab5c operates semi-independently from the other isoforms by promoting AMAP1-PRKD2 complex formation to enhance a growth factor-stimulated β1 integrin recycling pathway that regulates cancer cell invasion [54, 55].

Several experiments of membrane trafficking inhibition in different cell types reduce cell migration. However, the molecular mechanisms and pathways involved are still elusive [44]. In this study, we identified the CD93 cytotail as an essential domain for the spatiotemporal trafficking of CD93 during EC spreading and migration. Indeed, as happens upon Rab5c function depletion, we observed in a wound-healing assay that CD93 lacking of its cytotail is localized to the perinuclear region and not to the leading edge of migrating cells where F-actin is in active polymerization to form lamellipodia. These results are interesting not only because at the migrating front CD93 interacting with MMRN2 modulates β1 integrin activation, but also because the cytoplasmic domain of CD93 contains a consensus motif for the binding of Cbl, an adapter protein implicated in cell adhesion, organization of the actin cytoskeleton, and migration [12]. However, future work will determine if, by interacting with signaling protein(s), the CD93 cytotail triggers additional biochemical signal(s) promoting cell spreading and migration.

## Conclusions

In conclusion, our data, together with the fact that in the plasma membrane CD93 requires ongoing recycling and maintenance of an appropriate diffusional environment for its activity [56, 57], help to clarify the retrieval and recycling pathway of the CD93/MMRN2/β1 integrin complex during cell adhesion and migration, opening up new possibilities to modulate vascular physiology in human health and disease.

## Additional file

Additional file 1: **Figure S1.** Small vesicles containing CD93 colocalize with caveolin-1 in early spreading ECs. **Figure S2.** Disruption of the actin cytoskeleton impairs CD93 intracellular trafficking. **Figure S3.** Microtubule cytoskeleton disruption has no effects on CD93 intracellular trafficking. **Figure S4.** CD93 trafficking does not depend on transport from or to the Golgi complex. **Figure S5.** CD93 is colocalized with MMRN2 in spreading HDBECs. **Figure S6.** CD93 colocalizes strongly with Rab5 and slightly with Rab11, but not with Rab7. **Figure S7.** Rab5c is the predominant isoform in HUVECs. **Figure S8.** Rab5c regulates CD93 recycling to the cell surface. **Figure S9.** β1 integrin protein levels decrease at the leading edge of migrating Rab5c-silenced ECs. **Figure S10.** In CD93 silenced ECs, large Rab5c^+ve^ vesicles form regularly. (PDF 2570 kb)

## Data Availability

The data generated during this study are included in this article and its supplementary information files are available from the corresponding authors on reasonable request.

## Abbreviations

CTLD: C-type lectin-like domain
DIC: differential interference contrast
EC: endothelial cell
ECM: extracellular matrix
ERM: Ezrin/Radixin/Moesin
HDBEC: human dermal blood endothelial cell
HUVEC: human umbilical vein endothelial cell
MMRN2: Multimerin-2
TGN: *Trans*-Golgi network
wdc: white dot colocalization
