# Supplementary material for: The small GTPase Rab5c is a key regulator of trafficking of the CD93/Multimerin-2/β1 integrin complex in endothelial cell adhesion and migration
Source: Cell Commun Signal. 2019 May 28;17:55. doi: 10.1186/s12964-019-0375-x (PMC6537425; doi:10.1186/s12964-019-0375-x)
Supplement: Supplementary file 1 — Figure S1. Small vesicles containing CD93 colocalize with caveolin-1 in early spreading ECs. Figure S2. Disruption of the actin cytoskeleton impairs CD93 intracellular trafficking. Figure S3. Microtubule cytoskeleton disruption has no effects on CD93 intracellular trafficking. Figure S4. CD93 trafficking does not depend on transport from or to the Golgi complex. Figure S5. CD93 is colocalized with MMRN2 in spreading HDBECs. Figure S6. CD93 colocalizes strongly with Rab5 and slightly with Rab11, but not with Rab7. Figure S7. Rab5c is the predominant isoform in HUVECs. Figure S8. Rab5c regulates CD93 recycling to the cell surface. Figure S9. β1 integrin protein levels decrease at the leading edge of migrating Rab5c-silenced ECs. Figure S10. In CD93 silenced ECs, large Rab5c+ve vesicles form regularly. (PDF 2570 kb) [file 12964_2019_375_MOESM1_ESM.pdf]

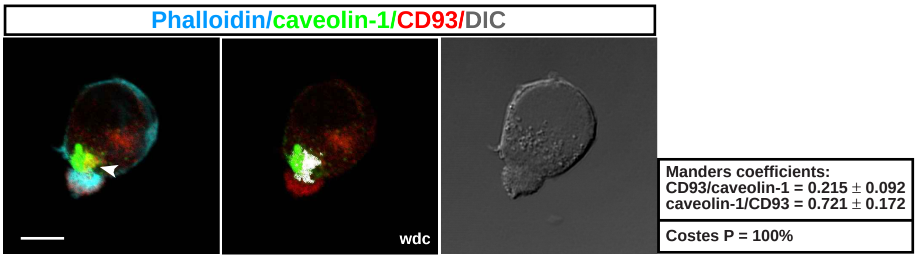

Supplementary Figure 1. **Small vesicles containing CD93 colocalize with caveolin-1 in early spreading ECs**. HUVECs were fixed during the initial phases of spreading and analyzed by immunofluorescence using phalloidin, anti-caveolin-1, and anti-CD93 antibodies. An arrowhead indicates small vesicles beneath the apical bud. Merged, wdc, and DIC images of stained cells are shown. Manders and Costes quantitative analyses of CD93 colocalization with caveolin-1 and vice versa are shown (n = 5 cells). Scale bar, 7 µm.

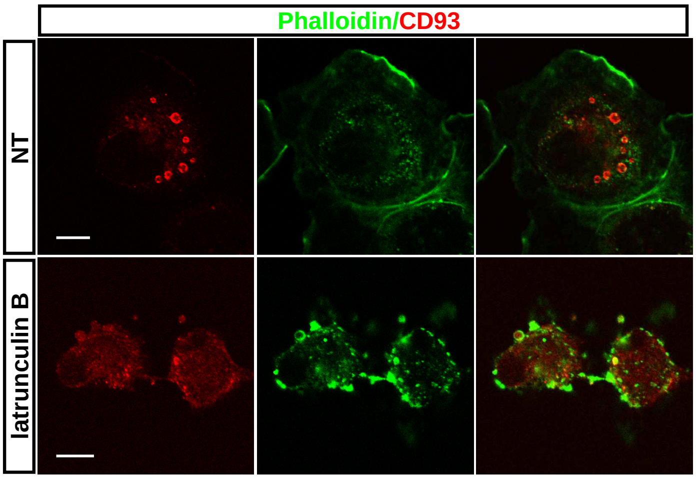

Supplementary Figure 2. Disruption of the actin cytoskeleton impairs CD93 intracellular trafficking. Subconfluent HUVECs were pretreated or not (NT) for 30 min with 0.5 µM latrunculin B, then were detached from the plate, resuspended in complete medium, and plated in the presence or not of latrunculin B. Cells were analyzed at late degrees of attachment to the substrate using anti-CD93 antibodies and phalloidin. Merged images are shown. Scale bars, 10 µm.

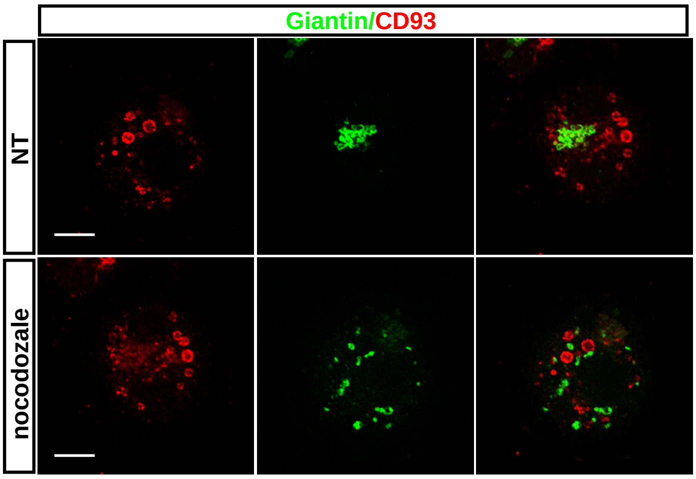

Supplementary Figure 3. Microtubule cytoskeleton disruption has no effects on CD93 intracellular trafficking. Subconfluent HUVECs were pretreated or not (NT) for 1 h with 20 µM nocodazole, then were detached from the plate, resuspended in complete medium, and plated in the presence or not of nocodazole. Cells were analyzed at late phases of spreading using anti-CD93 and anti-Giantin antibodies. Upon microtubule cytoskeleton depolymerization, the structural organization of the Golgi was dispersed throughout the cytoplasm. Merged images are shown. Scale bars, 10 µm.

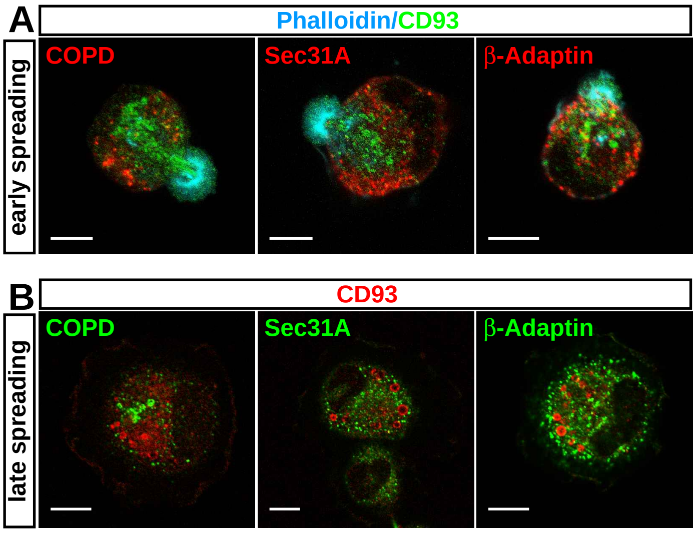

Supplementary Figure 4. CD93 trafficking does not depend on transport from or to the Golgi complex. Exponentially growing HUVECs were detached from the plate, resuspended in complete growth medium, plated on the substrate, and fixed at different time points of spreading. A: Cells were stained for F-actin, CD93, and COPD (a component of the COPI coat, which mediates retrograde transport from the Golgi), Sec31A (a component of the COPII complex, which promotes vesicle budding from endoplasmic reticulum), or β-Adaptin (a component of the AP1 and AP2 adapter complexes involved in the transport of clathrin-coated vesicles). Scale bars are 7 μm. B: Cells were stained for CD93 and for COPD, Sec31A, or β-Adaptin. Scale bars are 10 μm. Overlay of stained cells is shown.

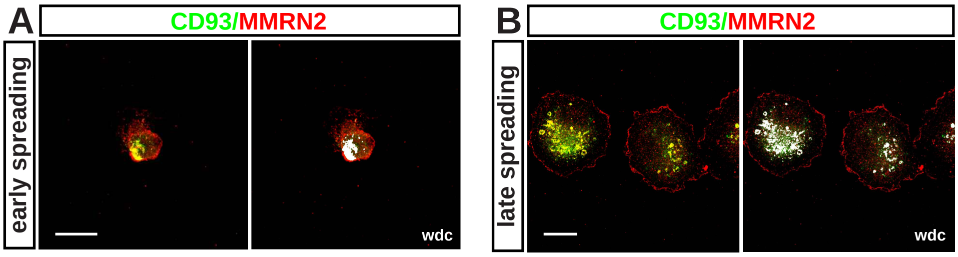

Supplementary Figure 5. CD93 is colocalized with MMRN2 in spreading HDBECs. Early (A) and late (B) spreading ECs were analyzed by confocal microscopy using anti-CD93 and anti-MMRN2 antibodies. Merged and wdc images are shown. Images were acquired using a Leica SP8 confocal microscope equipped and set as described in Fig. 3B. Scale bars, 20 µm.

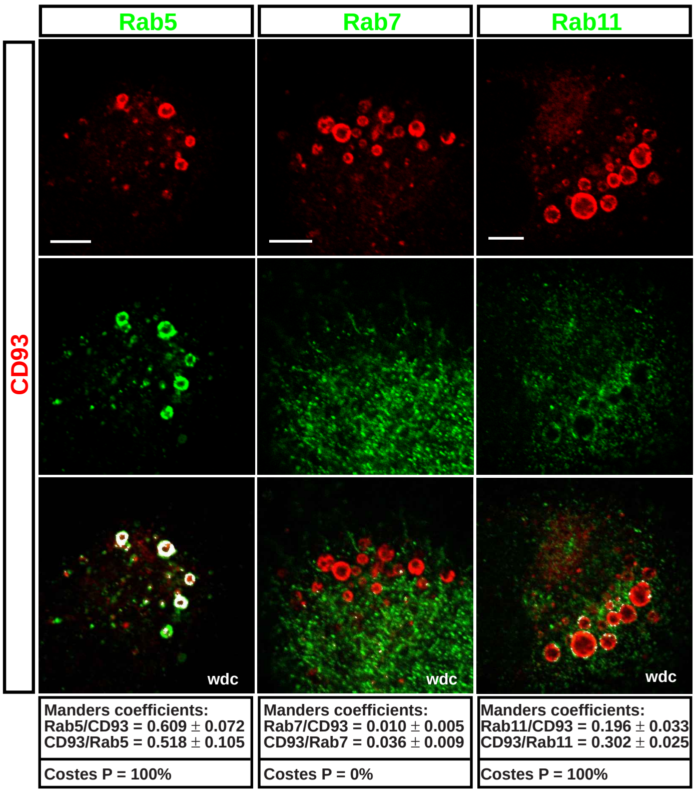

Supplementary Figure 6. CD93 colocalizes strongly with Rab5 and slightly with Rab11, but not with Rab7. Representative images of late spreading HUVECs analyzed by immunofluorescence using anti-CD93 and anti-Rab5, anti-Rab7, or anti-Rab11 antibodies. Wdc images are shown. Manders and Costes quantitative analyses of CD93 colocalization with Rab5, Rab7, or Rab11 and vice versa are shown (n = 10 cells). Scale bars, 6 µm.

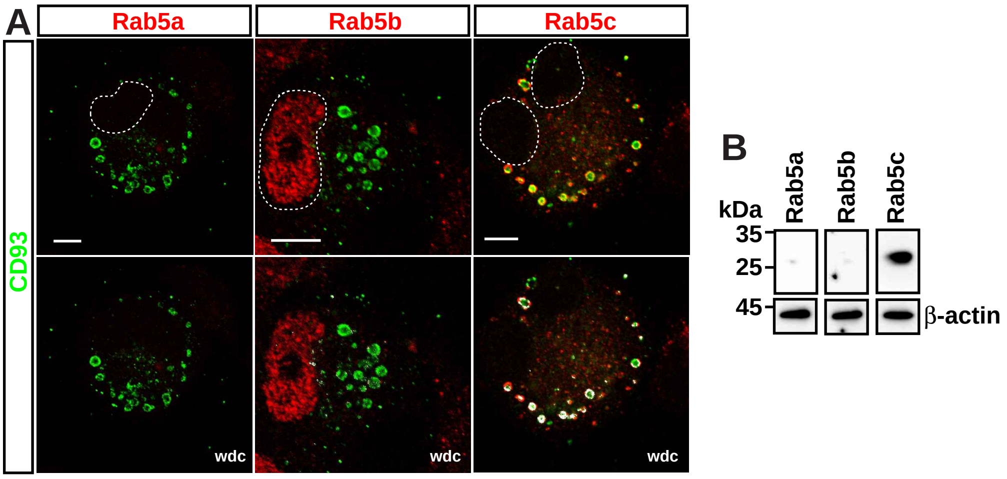

Supplementary Figure 7. Rab5c is the predominant isoform in HUVECs. A: Late spreading cells were imaged for immunofluorescence analysis using antibodies against CD93 and Rab5a, Rab5b or Rab5c. Nuclear staining for Rab5b is to be considered an unspecific signal. Merged and wdc images of stained cells are shown. Dotted lines indicate nucleus boundaries. Scale bars, 8 μm. B: Cell lysates from exponentially growing ECs were analyzed by Western blotting using antibodies raised against Rab5a, b, or c isoforms. Anti-β-actin antibodies were used to confirm equal loading.

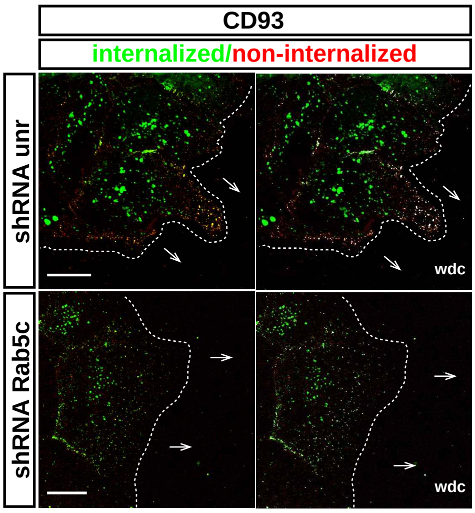

Supplementary Figure 8. **Rab5c regulates CD93 recycling to the cell surface**. HUVECs were transduced with lentiviral particles expressing unrelated (unr) or Rab5c (clone 33) shRNAs. After production of a scratch in the cell monolayer, cells were incubated with mouse anti-CD93 and Alexa Fluor-488 goat anti-mouse antibodies for 4,5 h in complete growth medium at 37° C. Then, cells were stained with an Alexa Fluor-568 donkey anti-goat antibody for 45 min in ice, fixed, and analyzed by confocal microscopy. Endocytosed CD93 is stained only with the secondary antibody (green), non-internalized CD93 with both the secondary and tertiary antibodies (yellow). Arrows indicate direction of migration and dotted lines indicate the migrating front. Merged and wdc images are shown. Same results were obtained when using the clone 31 for Rab5c knockdown. Scale bars, 20 μm.

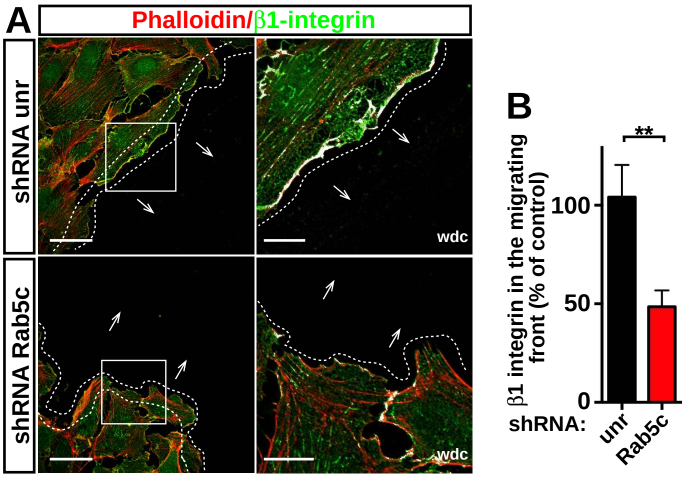

Supplementary Figure 9. β1 integrin protein levels decrease at the leading edge of migrating Rab5c-silenced ECs. A: Representative images of control (shRNA unr) and Rab5c-silenced (shRNA Rab5c, clone 33) HUVECs analyzed by confocal microscopy at 5 h after production of a double-sided scratch in the cell monolayer. Phalloidin and anti-β1 integrin antibodies were used to stain cells. Arrows indicate direction of migration and dotted lines indicate the migrating front. Overlay of stained cells is shown. Same results were obtained when using the clone 31 for Rab5c knockdown. Scale bars, 47 μm. Magnifications of the squared areas are shown as wdc images. In wdc images, scale bars are 15 μm. B: Quantification of β1 integrin along the migrating front areas indicated by dotted lines in A. Bars represent the percentage of fluorescence intensity of the control. Data are presented as the mean ± SD of three independent experiments (n = 6 different areas along the migrating edge). **$P < 0.01$; unpaired t-test.

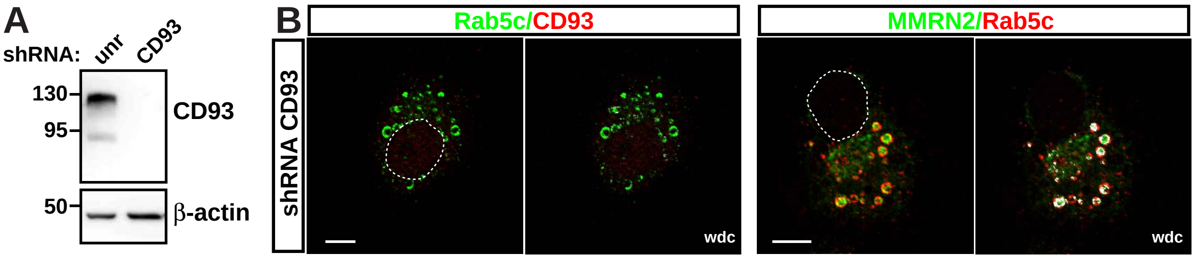

Supplementary Figure 10. **In CD93 silenced ECs, large Rab5c+ve vesicles form regularly**. HUVECs were transduced with lentiviral particles expressing unrelated (unr) or CD93 shRNAs. A: Cell lysates from shRNA expressing ECs were analyzed by Western blotting using antibodies against CD93. Anti-β-actin antibodies were used to confirm equal loading. B: Immunofluorescence analysis of CD93 knockdown cells imaged using antibodies against CD93 and Rab5c (left panels) or MMRN2 and Rab5c (right panels). Merged and wdc images of stained cells are shown. Dotted lines indicate nucleus boundaries. Scale bars, 8 μm.
